# Enhanced aerobic exercise performance in women by a combination of three mineral Chelates plus two conditionally essential nutrients

**DOI:** 10.1186/s12970-017-0199-2

**Published:** 2017-11-13

**Authors:** Robert A. DiSilvestro, Staci Hart, Trisha Marshall, Elizabeth Joseph, Alyssa Reau, Carmen B. Swain, Jason Diehl

**Affiliations:** 1 Human Nutrition, The Ohio State University, Columbus, OH 43210 USA; 2 Kinesiology, The Ohio State University, Columbus, OH 43210 USA; 3 OSU Sports Medicine, The Ohio State University, Columbus, OH 43210 USA

**Keywords:** Minerals, Conditionally essential nutrients, Running, Stationary biking, Step test

## Abstract

**Background:**

Certain essential and conditionally essential nutrients (CENs) perform functions involved in aerobic exercise performance. However, increased intake of such nutrient combinations has not actually been shown to improve such performance.

**Methods:**

For 1 mo, aerobically fit, young adult women took either a combination of 3 mineral glycinate complexes (daily dose: 36 mg iron, 15 mg zinc, and 2 mg copper) + 2 CENs (daily dose: 2 g carnitine and 400 mg phosphatidylserine), or the same combination with generic mineral complexes, or placebo (*n* = 14/group). In Trial 1, before and after 1 mo, subjects were tested for 3 mile run time (primary outcome), followed by distance covered in 25 min on a stationary bike (secondary outcome), followed by a 90 s step test (secondary outcome). To test reproducibility of the run results, and to examine a lower dose of carnitine, a second trial was done. New subjects took either mineral glycinates + CENs (1 g carnitine) or placebo (*n* = 17/group); subjects were tested for pre- and post-treatment 3 mile run time (primary outcome).

**Results:**

In Trial 1, the mineral glycinates + CENs decreased 3 mile run time (25.6 ± 2.4 vs 26.5 ± 2.3 min, *p* < 0.05, paired t-test) increased stationary bike distance after 25 min (6.5 ± 0.6 vs 6.0 ± 0.8 miles, *p* < 0.05, paired t-test), and increased steps in the step test (43.8 ± 4.8 vs 40.3 ± 6.4 steps, p < 0.05, paired t-test). The placebo significantly affected only the biking distance, but it was less than for the glycinates-CENs treatment (0.2 ± 0.4. vs 0.5 ± 0.1 miles, p < 0.05, ANOVA + Tukey). The generic minerals + CENs only significantly affected the step test (44.1 ± 5.2 vs 41.0 ± 5.9 steps, p < 0.05, paired t-test) In Trial 2, 3 mile run time was decreased for the mineral glycinates + CENs (23.9 ± 3.1 vs 24.7 ± 2.5, *p* < 0.005, paired t-test), but not by the placebo. All changes for Test Formula II or III were high compared to placebo (1.9 to 4.9, Cohen’s D), and high for Test Formula II vs I for running and biking (3.2 & 3.5, Cohen’s D).

**Conclusion:**

In summary, a combination of certain mineral complexes plus two CENs improved aerobic exercise performance in fit young adult women.

## Background

Micronutrient functions are needed for aerobic exercise. Therefore, aerobic performance should be helped by optimal intake of these nutrients including trace minerals. For instance, iron, even apart from its role in hemoglobin, affects aerobic energy metabolic pathways through functions in enzymes and cytochromes [1]. Another trace mineral, copper, is part of cytochrome c oxidase, the terminal enzyme in aerobic energy metabolism [1]. Other copper enzymes work against oxidative stress [1], which contributes to exercise-induced fatigue [2]. A third trace mineral, zinc, could affect aerobic exercise performance in a variety of ways: indirect antioxidant actions [3–5], a cofactor role in carbonic anhydrase that eliminates carbon dioxide [6, 7], a cofactor role in lactate dehydrogenase [6], and an activator of enzymes in energy metabolism [6].

For active young adult women, intake of these 3 minerals may often fall below optimal amounts. For iron, it well documented that exercising women often get a degree of deficiency [8]. Severe deficiency causes anemia, but even milder deficits could affect energy metabolism [9]. For copper, in young adult women, supplemental copper has improved copper function [10]. For zinc, in one USA diet survey, for about 40% of the women, intake fell below the RDA [11]. Moreover, the zinc RDAs may not even be set high enough [12, 13]. Multiple studies [1, 12–15] find low intake of zinc in active people. This low intake especially applies to participants in sports that need weight control, people who avoid animal products, and people eating high carbohydrate, low fat and protein diets. Furthermore, exercise training may raise copper and zinc requirements [14, 16, 17]. This situation may not be fixed easily by all multi-vitamin-mineral supplements since many use zinc and copper oxide, which are not the best absorbed forms [4].

On a related note, increased zinc or copper intake might benefit exercise performance even in non-deficient situations. In a controlled feeding study, eating above the copper RDA improves cycling performance and muscle cytochrome c oxidase versus RDA intake [18]. For zinc, in a rat study [19], plasma lactate level after swimming is highest in a low zinc diet group, medium in a zinc adequate group, and lowest in a zinc supplemented group. In similar work, moderately high zinc intake can raise lung levels of glutathione, an antioxidant, in swim trained rats [5].

Besides essential minerals, conditionally essential nutrients (CENs) also hold relevance to exercise. These molecules are both made by the body and eaten [1]. Traditionally, intake has been studied mostly for people with health problems i.e. [20]. However, in healthy rats or mice [21, 22], supplementation with the CEN carnitine delays exercise-induced fatigue. In humans, supplementing this same agent can influence muscle recovery, which can enhance training effects on exercise performance [23]. Carnitine can theoretically affect exercise performance and recovery via a role in fat oxidation for energy [24] and possibly via an anti-ischemic effect [25]. Another CEN, phosphatidylserine, could affect exercise performance by neuroendocrine effects, enzyme cofactor functions, and anti-inflammatory actions [26, 27]. Supplementation with this CEN influences perceived fatigue during exercise [28]. In addition, phosphatidylserine supplementation can extend time to exhaustion in a long biking session in active males [29]. The effects on other types of exercise measures in other types of exercisers await further research.

A new study tested the following hypothesis: increased intake of three trace minerals in well absorbed forms, plus two CENs, will improve aerobic exercise performance in recreationally trained, young adult women. The primary outcome was 3 mile runtime; secondary outcomes were distance covered in 25 min stationary biking and step number in a 90 s step test, both done shortly after the run. Positive results would show that increased trace mineral intake and/or CENs can improve aerobic exercise performance in aerobically active women. Later studies can further define the minimal combination of the 5 nutrients needed to produce the effects.

## Methods

### Participants

Subjects were non-smoking female, aged 18–30, with no injuries that restricted the ability to do aerobic exercise, and no acute major health problems or chronic conditions that affect energy metabolism or oxidative stress (ie anemia, diabetes, and cancer). Subjects were recreationally, aerobically trained in that they were not competitive athletes, but had regularly done aerobic exercise training at least120–180 min per week for at least 6 mo, with at least 2 days per week consisting of runs of 3 miles or more. All of these inclusion/exclusion criteria were evaluated based on answers to questions on an eligibility questionnaire. The subjects selected for study were instructed to maintain their current dietary and exercise practices.

### Procedures-trial 1

Subjects underwent exercise performance tests before and after a supplement intervention of 1 mo (*N* = 42). Subjects were randomly assigned to a supplement intervention in a double blind fashion. For Trial 1, subjects took a powder mixed by the research team. This team obtained the nutrients directly from suppliers who reported a third party analysis. The subjects were provided with a measuring spoon to add one serving to a beverage consumed just before or after a meal. The subjects could select the beverage, but it had to be one that they already consumed regularly. Low Calorie soft drinks or water + sweetener were the most common choices. Subjects split the daily dose into 2 servings. Assignments were as follows (*n* = 14/group):

#### Placebo (corn starch)

Test Formula I: 36 mg iron as ferrous sulfate, 2 mg copper as copper gluconate, 15 mg zinc as zinc gluconate, 2 g carnitine as carnitine tartrate (Carnipure® donated by Lonza, Basel, Switzerland), and 400 mg phosphatidylserine (SerinAid® donated by Chemi Nutra, Austin, TX).

Test Formula II: 36 mg iron as iron bisglycinate (Ferrochel® donated by Albion), 2 mg copper as copper glycinate (Albion), 15 mg zinc as zinc glycinate (Albion), 2 g carnitine as Carnipure® (Lonza), and 400 mg phosphatidylserine as SerinAid® (Chemi Nutra).

Exercise testing consisted of the following:

- Time for a 3 mile run (also used for Trial 2)
- Distance covered in 25 min of stationary cycling in the manual mode, done after a post-run walk for 200 m in about 1 min 30 s; the biking test used a resistance level determined on a day prior to the testing. If during the first testing, the subject felt the resistance setting was too hard or too easy, it was changed once. During the second testing, the times used at each resistance were repeated.
- A 90 s step test done about 1.5 min after the cycling; subjects walked during the 1.5 min recovery; results were total steps completed with alternating lead legs; the step test was done on a permanent bleacher area near the running track (step height of 41 cm).

### Procedures-trial 2

A new group of subjects (*N* = 34) was examined to look at run result reproducibility and a half dose of Carnipure® carnitine (1 g/day). Exercise testing consisted of time in a 3 mile run done before and after a 1 mo supplement intervention. For this Trial, subjects were given capsules. The minerals were combined into one capsule by Albion; the carnitine and phosphatidylserine capsules were purchased commercially. All products, including the purchased ones, had independent analysis of the contents. Random assignments were as follows (*n* = 17/group):

#### Placebo (corn starch)

Test Formula III-same as II, but with half the Trial 1 carnitine dose (capsules from Now Foods, Bloomingdale, IL), and phosphatidylserine as Cogni-PS**® (**Jarrow Formulas, Los Angeles, CA).

### Statistical analyses

Data are presented as mean or mean change ± SD. For the change data, 95% confidence intervals are included in the figure legends. Confidence intervals were calculated at http://www.fon.hum.uva.nl/Service/CGI-Inline/HTML/Statistics/Student_t_Test.html. For other statistical analysis, significance was set at *p* < 0.05. For each measure, for each of the treatment groups, post-intervention results were compared to pre-intervention results by paired t-test (http://www.fon.hum.uva.nl/Service/Statistics/Student_t_Test.html). For further statistical analysis, the results for each measure were expressed as the change in values (post intervention value – pre-intervention value). For Trial 1, where indicated, the changes in Test Formula II were compared to the changes in either of the other 2 groups by 1 way ANOVA + Tukey multiple comparison test (http://statistica.mooo.com/OneWay_Anova_with_TukeyHSD). For Trial 2, Test Formula III changes were compared to placebo by unpaired t-test (http://www.fon.hum.uva.nl/Service/CGI-Inline/HTML/Statistics/2Sample_Student_t_Test.html). For all changes in values, size effects were evaluated using Cohen’s D values using: http://www.socscistatistics.com/effectsize/Default3.aspx.

## Results

**Fig. 1:**
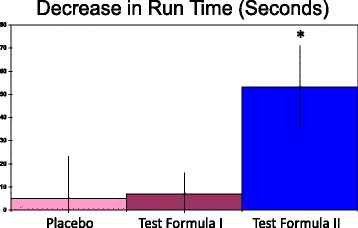
Decrease in run time after each of three treatments. Test Formula I contained generic minerals. Test Formula II contained glycinate minerals. Run times are mean decreases in min ± SD. The 95% confidence intervals for placebo, Test Formula 1, and Test Formula II were ±9.1, 5, and 9.4 respectively. *p < 0.05 versus either of the other two treatments

The data sets reported here were complete and did not exclude any data. Based on placebo data from Trial 1, the primary outcome, running times, showed good reliability based on a paired t-test >0.05, an intraclass correlation coefficient of 0.97 and a coefficient of variation of 3.6%. Test Formula II produced a substantial, statistically significant mean decrease in 3 mile run time of 54 s (*p* < 0.05, paired t-test, Table 1). When the data was expressed as the decrease in run times (Fig. 1), a 95% confidence interval did not come close to crossing 0 for Test Formula II, but did cross 0 for the placebo. For Test Formula I, the 95% confidence interval just missed crossing 0. The change for Test Formula II differed significantly from that produced by placebo or Test Formula I (Fig. 1). The size effects for Test Formula II were large compared to the other two groups (Cohen’s D of 2.7 vs placebo and 3.2 vs Test Formula I).

**Table 1:** Run times pre- and post-treatments

	Placebo	Test Formula I	Test Formula II
Run-Pre	26.6 ± 3.3	26.8 ± 3.5	26.5 ± 2.3
Run-Post	26.5 ± 3.3	26.6 ± 3.4	25.6 ± 2.4*

Test Formula I contains the generic minerals. Test Formula II contains the glycinate minerals. Run times are means of minutes ± SD. *p < 0.05 versus pre-value, paired t-test, two tailed

**Fig. 2:**
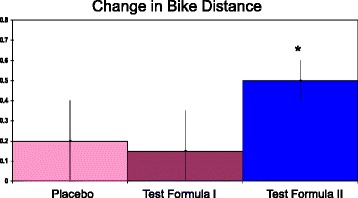
Increase in stationary bike distance after each of three treatments. Test Formula I contained generic minerals. Test Formula II contained glycinate minerals. Distances are mean increases in miles ± SD. The 95% confidence intervals for placebo, Test Formula 1, and Test Formula II were ±0.12, 0.12, and 0.05 respectively. *p < 0.05 versus either of the other two treatments

For the biking test, distance covered was increased significantly by both placebo and Test Formula II based on paired t-test (Table 2). Thus, reliability for this secondary outcome was not as strong as for the primary outcome. Even so, when the data was expressed as the change in distance (Fig. 2), the 95% confidence interval was just outside crossing 0 for the placebo, and touched 0 for Test Formula I. In contrast, the 95% confidence interval did not come close to crossing 0 for Test Formula II. The mean change for the bike distance was greater for the Test Formula II vs the placebo or Test Formula I (Fig. 2). The size effects for Test Formula II were large compared to the other two groups (Cohen’s D of 1.9 vs placebo and 3.5 vs Test Formula I).

**Table 2:** Stationary bike distance covered pre- and post-treatments

	Placebo	Test Formula I	Test Formula II
Bike-Pre	6.2 ± 0.8	6.1 ± 1.1	6.0 ± 0.8
Bike-Post	6.5 ± 0.9*	6.3 ± 1.1	6.5 ± 0.6*

Bike distances are mile means ± SD. *p < 0.05, versus pre-value, paired t-test, two tailed

**Fig. 3:**
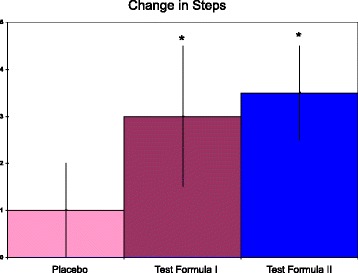
Increase in steps after each of three treatments. Test Formula I contained generic minerals. Test Formula II contained glycinate minerals. Step changes are mean increases in steps ± SD. The 95% confidence intervals for placebo, Test Formula 1, and Test Formula II were ±0.5, 0.8, and 0.5 respectively. *p < 0.05 versus placebo, but not versus each other (ANOVA + Tukey multiple comparisons)

Based on placebo data from Trial 1, the secondary outcome of the step test, showed good reliability based on a paired t-test >0.05, an intraclass correlation coefficient of 0.98 and a coefficient of variation of 3.2%. Test Formula I produced an increase that was significant by a one-tailed, paired t-test (*p* < 0.05, Table 3). Test Formula II treatment produced an increase that was strongly significant by a two-tailed, paired t-test (*p* < 0.005, Table 3). When the data was expressed as the change in steps (Fig. 3), a 95% confidence interval did not cross 0 for Test Formula I or II and was just outside crossing 0 for the placebo. The average change for the step test was greater for the both Test Formula groups versus placebo, but not versus each other (p < 0.05 versus placebo, 1 way ANOVA + Tukey multiple comparisons, Fig. 3). The size effects for Test Formula II were large compared to placebo, but small versus Test Formula I (Cohen’s D of 2.5 vs placebo and 0.4 vs Test Formula I).

**Table 3:** Step test pre- and post-treatments

	Placebo	Test Formula 1	Test Formula II
Steps-Pre	40.7 ± 7.0	41.0 ± 5.9	40.3 ± 6.4
Steps-Post	42.3 ± 6.4	44.1 ± 5.2*	43.8 ± 4.8**

Steps are number of step up means ± SD. *p < 0.05 versus pre-value, paired t-test, one tailed; **p < 0.005 versus pre-value, paired t-test, two tailed

**Fig. 4:**
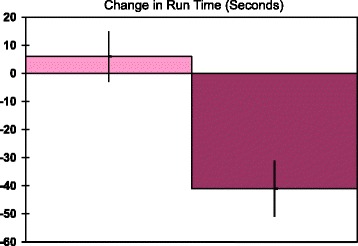
Change in run time after each of two treatments. Run time changes are means ± SD; The 95% confidence intervals for placebo, Test Formula 1, and Test Formula III were ±4.1 and 4.3 respectively. *p = 0.001, unpaired t-test, two tailed

A new group of subjects was examined to look at reproducibility of the run results and to test a lower dose of carnitine (Trial 2). Test Formula III, but not placebo, significantly decreased run time by paired t-test (*p* < 0.005, Table 4). The mean decrease was 41 s, which differed from placebo to a highly significant degree by unpaired t test (*p* = 0.001, Fig. 4). The mean time for the placebo actually increased, though this was not significant by paired t-test (Table 4). However, for the change in run time, the 95% confidence interval did not cross 0 for the placebo’s increase in time (Fig. 4). For the Test Formula III decrease in run time, the 95% confidence interval did not come close to 0. Also, the size effects for Test Formula III were large compared to placebo (Cohen’s D of 4.9).

**Table 4:** Run times pre- and post-treatments, Trial 2

	Placebo	Test Formula III
Run-Pre	23.2 ± 2.7	24.7 ± 2.5
Run-Post	23.4 ± 2.9	23.9 ± 3.1*

Run times are means ± SD; Test Formula III (half the carnitine dose of Test Formula I and II). *p < 0.005 versus pre-value, paired t-test, two tailed

## Discussion

The results of this study demonstrated that a combination of essential and conditionally essential micronutrients can strongly improve aerobic exercise performance. The particular nutrient combination tested here was chosen partly in light of the subjects studied, namely young adult women who are recreationally trained in aerobic exercise. For other types of subjects, the micronutrient mixture used here may or may not show the same efficacy as seen in the present study. Also, it is not known if all 5 micronutrients given here were needed for the effect. However, what the current study does shows is that aerobic exercise performance can respond to some type of increased micronutrient intake.

Replacement of the mineral glycinates with more standard mineral complexes restricted two of the three positive exercise effects. One of these substitutions was copper gluconate for copper glycinate. Copper gluconate has not shown effects in two non-exercise studies [30, 31]. In contrast, in a number of studies i.e. [10, 32, 33], copper glycinate has changed parameters such as copper enzyme activities and oxidized LDL readings. Zinc gluconate was also substituted for zinc glycinate. The former has produced effects in some studies i.e. [34], but not in some others i.e. [35]. In two studies, zinc glycinate has shown better bioactivity than zinc gluconate [35, 36]. In the one other substitution, ferrous sulfate was used instead of ferrous bisglycinate. The former is considered a standard form of iron for supplementation and has been effective [4]. The glycinate form of iron has shown a little better performance under some circumstances i.e. [4, 37–39] and may be better tolerated [4, 39]. Also, in a small unpublished study (DiSilvestro, RA), the 3 mineral glycinates used in this study increase plasma readings for the iron protein ferritin better than a combination of 3 other forms of the same minerals.

This study’s effects occurred in response to a sustained intake of nutrients rather than an acute pre-workout intake. However, the sustained ingestion was not tremendously long (1 mo). This timeframe was chosen because copper, iron and zinc nutritional status can all change in response to 1 mo or less of increased intake i.e. [3, 4, 38, 40, 41]. Similarly, carnitine supplementation can change metabolic responses to exercise in mice or humans in 1–3 weeks [21, 23]. Also, 10 day supplementation with phosphatidylserine alters cortisol response to exercise [27].

The exercise portion of the Trial 1was designed to produce three fatigue points in under an hour. This same exercise protocol was used in two previous studies from our group [42, 43]. The two previous studies examined an acute carbohydrate intervention before and during the exercise. In those cases, no effects were seen for the 3 mile run, but improvements were seen in the two subsequent exercise segments. A simple explanation for the previous results could be that substantial glycogen depletion had to start occurring before the intervention effect begins. In the present study, in both trials, an effect was seen in the run time results. Thus, in the present intervention, some mechanisms differed from those seen with the carbohydrate interventions.

In Trial 2, a good effect was seen for run time, though the mean decrease fell below that of Trial 1. This might have resulted simply from random variation. Another possibility was that changing the type of phosphatidylserine between trials diminished effectiveness to some degree. Another possible issue was that the daily carnitine dose was halved from 2 g to 1 g. However, this change probably exerted no effect. In a previous study, 2 g of carnitine was above a saturating dose for plasma levels [44]. Also, in an exercise recovery study with carnitine tartrate, a 1 and 2 g carnitine dose gave the same effects [45].

## Conclusions

In summary, this study showed that a combination of micronutrients can improve aerobic exercise performance in one set of circumstances. These findings justify further research on various combinations of micronutrients on exercise performance.

## Abbreviations

CENs: Conditionally essential nutrients
